# Poly(styrene sulfonic acid)-Grafted Carbon Black Synthesized by Surface-Initiated Atom Transfer Radical Polymerization

**DOI:** 10.3390/molecules28104168

**Published:** 2023-05-18

**Authors:** Artavazd Kirakosyan, Donghyun Lee, Yoonseong Choi, Namgee Jung, Jihoon Choi

**Affiliations:** 1 Department of Materials Science and Engineering, Chungnam National University, 99 Daehak-ro, Yuseong-gu, Daejeon 34134, Republic of Korea; 2 Graduate School of Energy Science and Technology (GEST), Chungnam National University, 99 Daehak-ro, Yuseong-gu, Daejeon 34134, Republic of Korea

**Keywords:** poly(styrene sulfonic acid), carbon black, ATRP, nanocomposites, O_2_ plasma

## Abstract

Owing to their excellent electrical conductivity and robust mechanical properties, carbon-based nanocomposites are being used in a wide range of applications and devices, such as electromagnetic wave interference shielding, electronic devices, and fuel cells. While several approaches have been developed for synthesizing carbon nanotubes and carbon-black-based polymer nanocomposites, most studies have focused on the simple blending of the carbon material with a polymer matrix. However, this results in uncontrolled interactions between the carbon filler and the polymer chains, leading to the agglomeration of the carbon filler. Herein, we report a new strategy for synthesizing sulfonated polystyrene (PSS)-grafted carbon black nanoparticles (NPs) via surface-initiated atom-transfer radical polymerization. Treatments with O_2_ plasma and H_2_O_2_ result in the effective attachment of the appropriate initiator to the carbon black NPs, thus allowing for the controlled formation of the PSS brushes. The high polymeric processability and desirable mechanical properties of the PSS-grafted carbon black NPs enable them suitable for use in nonfluorinated-hydrocarbon-based polymer electrolyte membranes for fuel cells, which must exhibit high proton conductivity without interrupting the network of channels consisting of ionic clusters (i.e., sulfonic acid moieties).

## 1. Introduction

Carbon-based nanomaterials (i.e., fullerenes, carbon nanotubes (CNTs), graphene, and carbon black) have attracted significant attention owing to their excellent electrical and thermal conductivities as well as high mechanical strength. They are being used in a wide range of applications and devices such as electronic devices [1,2], electromagnetic wave (EM) interference shielding [3,4], and polymer electrolyte membrane fuel cells (PEMFCs) [5,6]. Carbon-based nanomaterials are also being employed extensively as the reinforcing components in polymer nanocomposites to enhance the properties of the flexible polymer media [7,8]. For example, CNT–polystyrene composites with significantly enhanced EM-wave-absorbing properties have been reported; these properties can be attributed to the percolation structure of the CNT networks at the microscopic level, which effectively increases the electrical conductivity of the networks at low CNT loading rates (of the order of a few wt%) [4]. Similarly, CNTs and graphene have been used as low-dimensional nanofillers to form a continuous high-thermal-conductivity phase within polymer nanocomposites for use in conventional electronic devices that require thermal management [9,10]. Lee et al. reported the electrical and thermal conductivity of multi-walled CNTs (MWCNTs) composites consisting of polydimethylsiloxane and MWCNTs with a different length to diameter ratio, in which considerably enhanced electrical and thermal properties were demonstrated for MWCNT composites with a high aspect ratio over the low aspect ratio. Additionally, they showed that the thermal conductivity of MWCNT composites increases along with the concentration of MWCNTs in the composite. However, most studies have focused on the simple blending of carbon-based nanomaterials within a polymer matrix, despite the importance of controlling the spatial arrangement or distribution of the nanofillers, which has a determining effect on the overall physical properties of the polymer nanocomposites [11,12]. For instance, Göldel et al. showed that the geometry of carbon-based nanoparticles (i.e., spherical carbon blacks and CNTs with a high aspect ratio) has a crucial role on the localization of carbon particles in the composite blends prepared via melt mixing of carbon materials with a poly(styrene acrylonitrile). Donchak et al. reported nanoarchitectured multi-wall carbon nanotubes (MWCNTs) with polymer for enhanced dispersion and improved mechanical properties of nanocomposites in comparison with non-modified fillers. MWCNTs were modified by multifunctional peroxide initiators of radical polymerization, which are able to form covalent bonds with CNTs and contain peroxide groups for formation of free radicals to bond macromolecules of polymer matrix via the chain transfer reaction [12]. Although several routes have been proposed to overcome the challenges associated with aggregation of the nanofillers owing to their surface functionalization in order to increase their chemical affinity, including various orientation/alignment processing techniques, the uniform distribution of carbon-based nanomaterials remains the key to increasing their practical applicability.

The uniform dispersion of nanofillers in nonfluorinated-hydrocarbon-based polymer electrolyte membranes (PEMs), which serve as the separators and electrolytes in PEMFCs for proton transport from the anode to the cathode is particularly important [10,13]. This is because the agglomeration of the nanofillers severely reduces proton conductivity by interrupting the network of channels consisting of ionic clusters (i.e., sulfonic acid moieties). Various inorganic materials, such as metal oxides, zirconium phosphates, clays, and heteropolyacids, have been used to increase the water uptake and mechanical strength of PEMs [14,15,16,17]. For example, Choi et. al. reported that multiwalled CNTs were blended with a perfluorinated-sulfonic-acid-based polymer (Nafion™) to produce mechanically reinforced polymer membranes for PEMFCs with improved stiffness and dimensional stability [14]. However, the aggregated CNTs hindered the effective transport of protons across the PEM. Furthermore, the device exhibited poor durability owing to the significant volume changes (expansion and shrinkage) that occurred during operation. Similarly, Tang et al. demonstrated that poly(phenylacetylenes) can wrap around MWCNTs at a molecular level in the process of in situ polymerizations of phenylacetylene [16]. The polymer nanocomposites prepared by wrapping the CNTs with polymer chains showed a high solubility in several solvents including tetrahydrofuran, toluene, chloroform, and 1,4-dioxane. Furthermore, Lou and coworkers showed that the addition reaction can effectively promote the covalent attachment of polystyrene, poly(3-caprolactone) or block copolymers of these two polymers to MWCNTs. In this reaction, the polymer chains were end-functionalized by 2,2,6,6-tetrame-thylpiperidine-1-oxyle (TEMPO) group that reacts with CNTs via the alkoxyamine-terminated precursors according to a radical mechanism. Polymer-grafted MWCNTs composites are easily dispersed in common solvents such as toluene and THF [17]. Thus, strong covalent bonds between the inorganic fillers and polymer chains are necessary to prevent phase segregation under harsh operating conditions.

In our prior study, we have demonstrated that the formation of polymer brushes on the nanoparticle (NP) surface can effectively control the structure formation and mechanical properties. In particular, a precise control of the grafting density and the molecular weight of grafted polymer chains enable the structural evolution from ‘ordered’ to ‘disordered’ and the mechanical transition from ‘hard-sphere-like’ to ‘polymer-like’ of particle solids [18,19,20,21]. Furthermore, toughening of particle solids highly depends on the degree of polymerization of grafted chains that exceeds a critical value as a consequence of the entanglement between surface-grafted polymer chains. These studies have demonstrated that the presence of polymer brushes can provide an innovative route for fabrication of the compliant and mechanically robust “matrix-free” particle films.

In this study, we show that polystyrene sulfonate (PSS) brushes can be covalently grafted onto commercial carbon black (Vulcan XC-72) to form durable composite materials for highly stable PEMs via surface-initiated atom-transfer radical polymerization (SI-ATRP) [18,19,20,21,22,23,24]. In particular, the functionalization of the carbon black is essential for forming covalent bonds between its surface and the initiator for the ATRP process. A combination of O_2_ plasma and H_2_O_2_ treatments provided the most efficient formation of O−C=O functional groups on the surface of the carbon black, resulting in a PSS-grafted carbon black composite with a high polymer/carbon (PSS:C) mass ratio. Therefore, we were able to endow carbon black with mechanical robustness and polymer-like processability. These properties make the obtained composite suitable for use in multifunctional PEMs such that the inorganic filler does not readily exhibit agglomeration.

## 2. Results and Discussion

**Figure 1:**
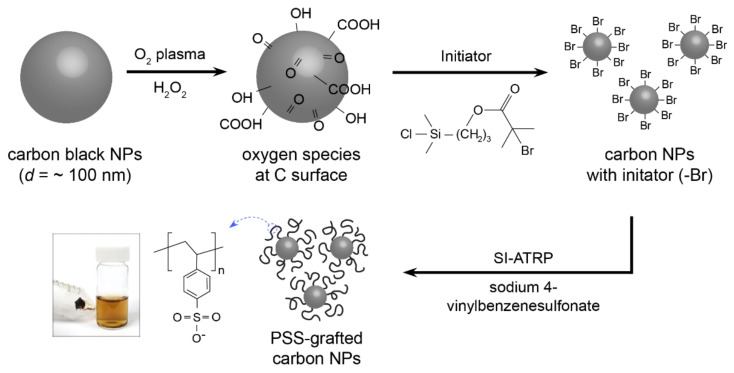
Schematic illustration of the formation of carboxyl groups, ATRP initiator functionalization, and surface–initiated ATRP process of sulfonated polystyrene on carbon black nanoparticles.

The PSS-grafted carbon black (PSS@C) nanoparticles (NPs) were produced by subjecting the pristine carbon black NPs to surface treatments and subsequently performing SI-ATRP to form PSS brushes on the surfaces of the functionalized NPs, as illustrated in Figure 1. First, the commercial carbon black NPs (diameter ~ 100 nm) were functionalized by O_2_ plasma and H_2_O_2_ treatments to introduce carboxylic groups (–COOH) on their surfaces. The O_2_ plasma treatment introduced several functional groups such as the C=O and ≡C–O–C≡ groups along with other similar oxygen-containing groups. The H_2_O_2_ treatment converted the C–O group into the –C–OH and –COOH groups, which can react with the end group (Si–Cl) of the initiator used (1-(chlorodimethylsilyl)propyl-2-bromoisobutyrate), thus anchoring the initiator molecules onto the surfaces of the carbon black NPs. Next, a typical ATRP process was performed using sodium sulfonated styrene (Na-SS) monomers to produce the PSS@C composite material [25,26].

**Figure 2:**
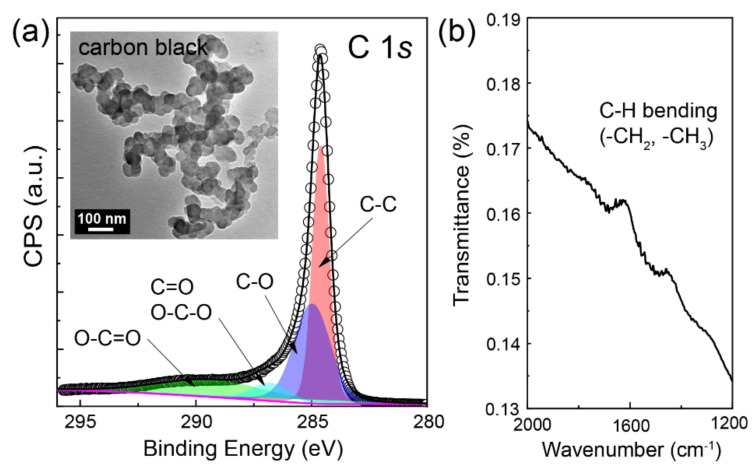
(**a**) C 1*s* XPS and (**b**) FTIR spectrum of pristine carbon black NPs. Inset shows the representative TEM image of carbon black NPs.

We investigated the nature of chemical bonds in the carbon materials using X-ray photoelectron spectroscopy (XPS) and Fourier-transform infrared spectroscopy (FTIR) (Figure 2). Figure 2a shows a representative high-resolution XPS spectrum of the pristine carbon black sample used. The C 1*s* peak could be deconvoluted into four subpeaks corresponding to the C–C, C–O, C=O (O–C–O), and O–C=O functional groups; the corresponding binding energies were 284.5, 285.9, 287.3, and 289.3 eV, respectively [27,28,29]. While a few minor functional groups, such as C=O and O–C=O, were responsible for the extremely weak shoulder peaks observed at higher binding energies, the C–C (284.5 eV) bond showed the strongest C 1*s* peak, indicating that the minor functional groups were present in negligibly low numbers on the surfaces of the carbon black NPs. Furthermore, the FTIR spectrum contained a band at 1430–1470 cm^−1^, which was related to (C–H) bending vibrations; these, in turn, were ascribable to the –CH_2_ and –CH_3_ groups (Figure 2b) [30]. All these functional groups are present naturally in pristine carbon materials. The additional band at 1621 cm^−1^ in the FTIR spectrum could be attributed to the moisture adsorbed onto the surface of the carbon black. Finally, the size of the pristine carbon NPs (~100 nm) was determined from a TEM image (Figure 2a, inset).

**Figure 3:**
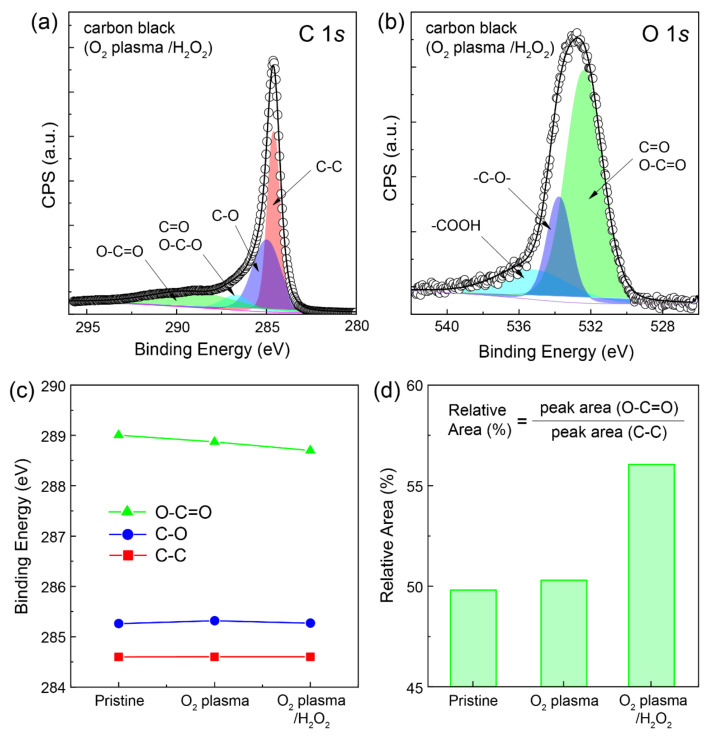
XPS spectra of (**a**) C 1*s* and (**b**) O 1*s* of the carbon black NPs with a post-treatment (O_2_ plasma/H_2_O_2_). (**c**) Binding energy of the characteristic functional groups (O−C=O, C−O, and C−C) in the C 1*s* XPS spectrum and (**d**) the relative area of the corresponding functional group (O−C=O) depending on the post−treatment of carbon black NPs.

Further investigation of the additional chemical reaction was performed for the carbon black samples subjected to the O_2_ plasma and H_2_O_2_ treatments (Figure 3). The high-resolution XPS C 1*s* and O 1*s* spectra of the carbon black samples after the O_2_ plasma and H_2_O_2_ treatments were measured (Figure 3a,b). Similar to the case for the pristine carbon black sample, peaks related to various functional groups such as C–C, C–O, C=O (O–C–O), and O–C=O were observed at almost identical positions; this was the case after both treatments (Figure 3c). However, the peak centered at 288.7 eV corresponding to the O-C=O group exhibits a slight decrease of 0.3 eV after the O_2_ plasma and H_2_O_2_ treatments, indicating changes in the chemical state of the carbon bonded to elemental oxygen. Furthermore, the relative O–C=O/C–C ratio, determined from the C 1*s* spectrum, obviously indicated an increase in oxygen-containing functional groups after the O_2_ plasma and H_2_O_2_ treatments (Figure 3d). Notably, the content of the –COOH group was significantly enhanced by the chemical reaction of H_2_O_2_ with the oxygen-containing functional groups such as C=O, O–C–O, and –C–O–. The XPS O 1*s* spectrum exhibits clearly peaks related to C=O/O–C–O, –C–O–, and –COOH at 532.0, 533.8, and 535 eV, respectively (Figure 3b) [29,31]. Furthermore, it was observed that the content of –COOH groups increased significantly owing to the reduction in the O–C–O group after the H_2_O_2_ treatment. This confirmed that the content of the oxygen-containing functional groups had increased; this is beneficial for the chemical bonding of the initiator for the ATRP process. From the XPS analysis, it was confirmed that the C and O contents of the pristine carbon black sample were 98.6% and 1.2%, respectively. After the pretreatment with the O_2_ plasma, these values were 95.5% and 3.4%, respectively. Finally, the oxygen content increased further, to 5.8%, after the H_2_O_2_ treatment.

**Figure 4:**
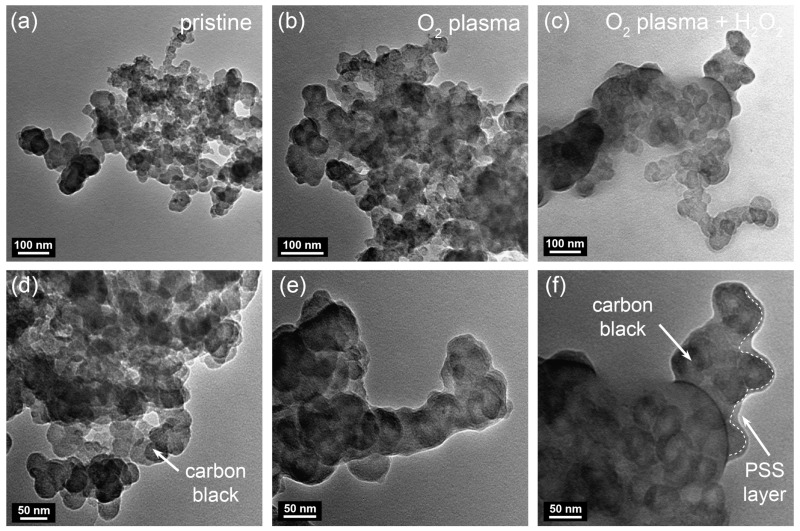
TEM images of the PSS-grafted carbon black NPs aggregates at (**a**–**c**) low and (**d**–**f**) high magnification using (**a**,**d**) pristine carbon, (**b**,**e**) O_2_ plasma-treated carbon, and (**c**,**f**) O_2_ plasma/H_2_O_2_ treated carbon. Dashed line on (**f**) indicates the interface between PSS layer and carbon black NPs.

**Figure 5:**
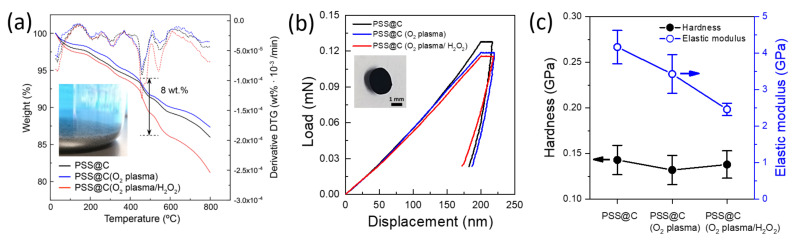
(**a**) Thermogravimetric analysis of PSS−grafted carbon black NPs for pristine (black), O_2_ plasma−treated (blue), and O_2_ plasma/H_2_O_2_ treated (red) carbon. Inset shows the appearance of PSS-grafted carbon black NPs (O_2_ plasma/H_2_O_2_) precipitated in methanol. Blue color of the solution is due to the presence of Cu ions used in the ATRP process. (**b**) Load−displacement curves obtained by nanoindentation and (**c**) the corresponding hardness and elastic modulus.

The PSS brushes were successfully grafted from the initiator onto the surfaces of the O_2_-plasma/H_2_O_2_-treated carbon black NPs via SI-ATRP. The TEM images showed that, after the SI-ATRP process, the shape and size of the carbon black NPs in the PSS@C composites were similar to those of the pristine carbon black NPs (Figure 4). However, a PSS layer was not observed on the pristine carbon black NPs after the ATRP process (Figure 4a,d); this was because the content of the oxygen-containing functional groups required to attach the initiator to the surfaces of the carbon black NPs was low. In contrast, the TEM images of the samples treated with the O_2_ plasma/H_2_O_2_ indicated the formation of a thin PSS layer on the surfaces of the carbon black NPs. In particular, a thin PSS layer of 4 ± 1.2 nm could be seen clearly in the case of the sample subjected to both the O_2_ plasma treatment and the H_2_O_2_ treatment, despite the duration of the ATRP process being the same (Figure 4f, dashed line). This trend is well matched with the increase in the PSS/carbon mass ratio (i.e., a weight loss of the samples in the temperature range from 430 to 500 °C) as determined by TGA (Figure 5a) [24,32]. Anchoring an ATRP initiator to the surfaces of carbon black NPs is challenging because of the absence of the appropriate functional groups that can ensure chemical bonding. However, the O_2_ plasma and H_2_O_2_ treatments together produced more reactive functional groups that promoted the anchoring of the ATRP initiator on the surfaces of the carbon black NPs. Thus, the resulting product has a higher content (~8 wt%) of PSS, exhibiting a thicker film.

To characterize the mechanical properties of the samples, such as their elastic modulus and hardness, ~100 μm-thick films of the samples were subjected to nanoindentation (Figure 5b,c). Typically, the carbon black NPs result in crack formation due to the residual stress during drying process, exhibiting a poor wettability with a coffee-ring effect (i.e., packed particles at the edge) induced by initial pinning and subsequent self-pinning of particles. This results in non-uniform mechanical properties and significant defect formation in the film. However, the inset of Figure 5b shows a crack-free film consisting of PSS-grafted carbon composites, in which the presence of the PSS layer at the surface of the carbon black NPs exhibits more polymer-like behavior. The mechanical characteristics of the PSS-grafted carbon black film were analyzed by nanoindentation. Figure 5b shows the representative load–displacement curves after the different treatments. The presence of PSS after the treatments had a noticeable effect on indent formation. Interestingly, the slope of the load–displacement curves decreased after both the O_2_ plasma treatment and the combined O_2_ plasma/H_2_O_2_ treatments. The average elastic modulus and hardness were determined from the load–displacement curves obtained at a strain rate of 0.05 s^−1^; 30 distinct indents were performed per sample using a Berkovich indenter (Figure 5c). While the hardness was independent of the treatment performed and lay in the 0.132–0.143 GPa range, the elastic modulus was reduced significantly, from 4.17 GPa (for the pristine carbon black NPs) to 3.43 GPa for the O_2_-plasma-treated sample and 2.46 GPa for the O_2_-plasma/H_2_O_2_-treated sample. This reduction in the elastic modulus is attributed to the presence of a significant amount of PSS, which has a lower elastic modulus than that of carbon black. In particular, the entanglement between the PSS chain segments plays a crucial role to enhance their mechanical properties. It also confirmed the presence of PSS brushes, which can increase the flexural strength and resilience of carbon black NPs against external mechanical strains.

**Figure 6:**
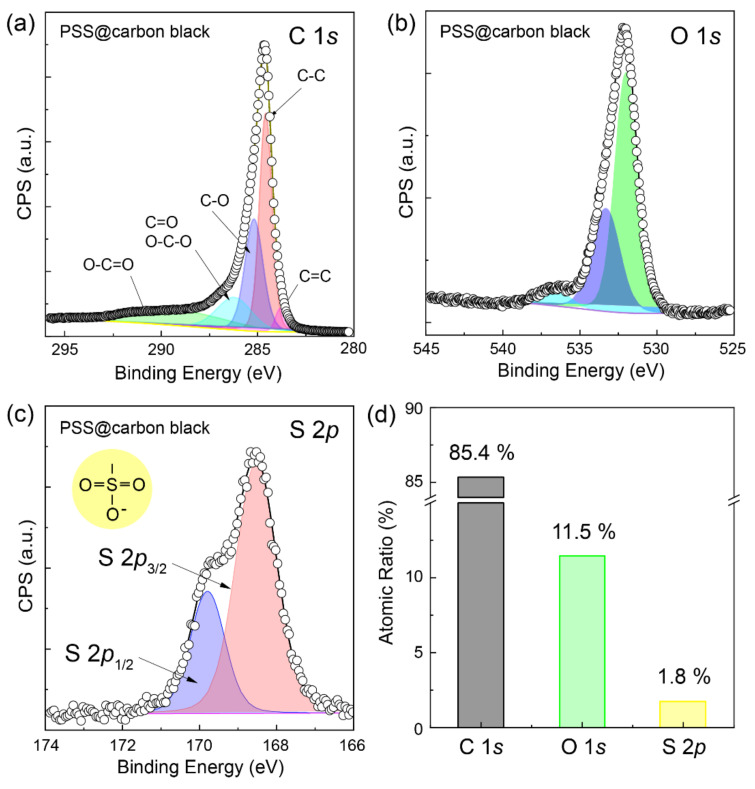
XPS spectra of (**a**) C 1*s*, (**b**) O 1*s*, and (**c**) S 2*p* of the PSS-grafted carbon black NPs with a post-treatment (O_2_ plasma/H_2_O_2_). (**d**) Atomic ratio of the corresponding elements (C, O, and S) in the PSS−grafted carbon black NPs.

The elemental contents and chemical states of the PSS-grafted carbon black NPs after the O_2_ plasma/H_2_O_2_ treatments were determined using high-resolution XPS C 1*s*, O 1*s*, and S 2*p* spectral analysis (Figure 6). The contents of C, O, and S in the sample were 85.4%, 11.5%, and 1.8%, respectively (Figure 6d). The XPS C 1*s* and O 1*s* spectra are similar to those of O_2_ plasma/H_2_O_2_ treated carbon NPs where C 1*s* and O 1*s* are fitted with multiple subpeaks centered at 284.5, 285.9, 287.3, and 289.3 eV corresponding to C–C, C–O, C=O (O–C–O), and O–C=O functional groups, and at 532.0, 533.8, and 535 eV corresponding to C=O/O–C–O, –C–O–, and –COOH, respectively. The XPS C 1*s* and O 1*s* spectra confirmed the characteristic features of the sulfonated polystyrene. In addition, the contents of the oxygen singly and doubly bound to carbon were also determined. The higher oxygen content compared to combined O_2_ plasma/H_2_O_2_ treated carbon (11.5% vs. 5.8%) could be attributed to the presence of the –SO_3_^−^ functional group (Figure 6a,b). The elemental sulfur of the –SO_3_^−^ functional group in the sulfonated styrene exhibited spin-orbit splitting to S 2*p*_1/2_ and S 2*p*_3/2_ at 169.8 and 168.5 eV, respectively; this is consistent with the literature [31,33]. These results confirmed that PSS brushes were formed successfully on the carbon black NPs via the SI-ATRP process, thus significantly enhancing their processability. Hence, this method is highly suited for producing crack-free carbon composites without having to blend filler materials with a polymeric base (Figure 5b).

## 3. Materials and Methods

### 3.1. Materials

Sodium sulfonated styrene (Na-SS ≥ 90%), copper (I) bromide (CuBr, 98%), copper (II) bromide (CuBr2, 99.999%), N,N,N′,N′′,N′′-pentamethyldiethylenetriamine (PMDETA, 99%) and diethyl ether (≥99%) were purchased from Sigma-Aldrich and used as received without any further purification unless explicitly stated. Toluene (99%), tetrahydrofuran (99%), dimethylformamide (DMF, 99.5%), and glacial acetic acid (99.7%) were purchased from Daejung Chemicals and Metals. Copper(I) bromide was purified by washing with glacial acetic acid and diethyl ether, and then filtered, dried, and stored under vacuum for further use. Atom transfer radical polymerization (ATRP) initiator 1-(chlorodimethylsilyl)propyl-2-bromoisobutyrate was synthesized by direct reaction of chlorodimethylsilane (98%) and allyl 2-bromo-2-methylpropionate (98%) in presence of Pt catalyst at room temperature as described in the Section 3.2.

### 3.2. Functionalization of Carbon Black Nanoparticles

First, carbon black (Vulcan XC-72; 50 mg) NPs was dispersed in DMF (10 mL) via tip sonication for 10 min. Oxygen plasma treatment was performed using a CUTE Plasma system (Femto Science Inc., Hwaseong, Republic of Korea). The samples were placed in the chamber and the treatment was conducted for 5 min in an oxygen plasma environment with an O_2_ flow rate (20 cm^3^/s). The electrical power of the oxygen plasma was set to 100 W (70 kHz). Hydrophobic carbon black powder becomes hydrophilic, and their wettability to deionized (DI) water increases significantly after O_2_ plasma treatment. This is direct evidence for the formation of oxygen-containing functional groups. For the H_2_O_2_ treatment, O_2_ plasma-treated carbon black NPs (50 mg) were boiled in a mixture of DI water (5 mL) and H_2_O_2_ (5 mL) solution for 20 min and dried under vacuum. After O_2_ plasma and H_2_O_2_ treatment, the dispersion of carbon black NPs was significantly increased in polar solvents such as DI water and DMSO.

### 3.3. Synthesis of 1-(Chlorodimethylsilyl) Propyl-2-bromoisobutyrate ATRP Initiator

1-(chlorodimethylsilyl) propyl-2-bromoisobutyrate is used as ATRP process initiator. The ATRP initiator was synthesized according to previously reported procedures based on hydrosilation of ally-2-bromoisobutyrate and chlorodimethylsilane using Karstedt’s catalyst [22]. Allyl-2-bromoisobutyrate (3.85 mL, 24.2 mmol) and excess amount of chlorodimethylsilane (18.12 mL, 145 mmol) were added into an oven-dried 100 mL round bottom flask equipped with a magnetic stirring bar. The flask was placed in an ice bath and stirred for 30 min. Karstedt’s catalyst (0.4 mL, 0.038 mmol) was added to the solution mixture dropwise. The reaction was allowed to proceed for 36 h at room temperature. Complete disappearance of allyl-2-bromoisobutyrate, and hence the completion of reaction, was confirmed by ^1^H NMR spectroscopy. The excess chlorosilane was removed under vacuum for 2 h. The obtained 1-(chlorodimethylsilyl) propyl-2-bromoisobutyrate was stored for further use.

### 3.4. ATRP of PSS-Grafted Carbon Black NPs

The ATRP procedure for the PSS-grafted carbon black NPs was performed according to previously published procedure [23,24,25,26]. First, carbon black NPs were functionalized with an alkyl halide initiator, 1-(chlorodimethylsilyl) propyl-2-bromoisobutyrate. The grafting density (ρ) was controlled to be high (>0.5 nm^−2^) using excess amounts of initiator. A mixture of initiator-functionalized carbon black NPs dispersed in DMF, a sulfonated styrene monomer (Na-SS), CuBr_2_, PMDETA ligands, and DI water in a Schlenk flask was sonicated for 30 min to form a homogeneous suspension. After three freeze–pump–thaw cycles, Cu (I)Br was added to the nitrogen-filled flask. The ATRP reaction was initiated by heating the solution in an oil bath (T = 70 °C). The molar ratio of the chemical components in a typical reaction was approximately Na-SS:CuBr:CuBr_2_:PMDETA at 2000:1:1:11. Polymerization was terminated by introducing air into the reaction flask after three days of reaction. The product was precipitated in tetrahydrofuran, collected by centrifugation at 6000× *g* rpm for 15 min, and dissolved in DI water to remove the unreacted reactants. This washing process was repeated until the Cu (II) catalyst was removed, as evidenced by the disappearance of its characteristic blue color. The final product was dried in a vacuum oven at 60 °C.

### 3.5. Characterization

The weight ratio of poly(styrene sulfonic acid) was determined using thermogravimetric analysis (TGA/DSC1, Mettler-Toledo) under a continuous flow of N_2_ gas at temperatures up to 800 °C at a heating rate of 10 °C/min. Transmission electron microscopy (TEM) images were acquired using a JEM 2010 electron microscope operating at 200 keV. X-ray photoelectron spectroscopy (XPS) spectra were collected using a Thermo Fisher Scientific/K-Alpha+ X-ray photoelectron spectrometer equipped with a monochromatic X-ray source from Al Kα (hν = 1486.6 eV). Fourier transform infrared (FTIR) spectra were obtained in the range 400–4000 cm^−1^ using a VERTEX 80 v Vacuum Infrared Spectrometer (Bruker, Ettlingen, Germany). For estimating the elastic modulus and hardness, nanoindentation experiments were performed using a KLA iMicro Nanoindenter equipped with a Berkovich indenter. The specimens were loaded to the maximum displacement of 200 nm at a constant indentation strain rate of 0.05/s.

## 4. Conclusions

Sulfonated-polystyrene-grafted carbon NPs were successfully prepared by subjecting carbon black NPs to a combination of O_2_ plasma and H_2_O_2_ treatments and then the SI-ATRP process. Specifically, PSS brushes could be formed on carbon black NPs by the surface treatment of the carbon black using O_2_ plasma and H_2_O_2_; the presence of the -COOH functional group is crucial for attaching the ATRP initiator and the subsequent formation of the PSS brushes. The PSS-grafted carbon black NPs exhibited enhanced mechanical strength (elastic modulus = 2.46 GPa, hardness = 0.143 GPa) and processability and could be produced without blending. Therefore, these carbon-based nanocomposites are highly suited for use in multifunctional PEMs as their properties are not adversely affected by agglomeration of the carbon filler.

## Data Availability

Not applicable.

## Sample Availability

Samples of the compounds are not available from the authors.
